# Avian leukosis virus in indigenous chicken breeds, China

**DOI:** 10.1038/emi.2015.76

**Published:** 2015-12-30

**Authors:** Xuan Dong, Peng Zhao, Bu Xu, Jianhua Fan, Fanfeng Meng, Peng Sun, Sidi Ju, Yang Li, Shuang Chang, Weifeng Shi, Zhizhong Cui

**Affiliations:** 1 College of Veterinary Medicine, Shandong Agricultural University, Taian 271018, Shandong Province, China; 2 Jiangsu Institute of Poultry Science, Yangzhou 225125, Jiangsu Province, China; 3 Institute of Pathogen Biology, Taishan Medical College, Taian 271000, Shandong Province, China

## 

**Dear Editor**,

Avian leukosis viruses (ALVs) can induce various tumors and cause production problems.^1^ Besides being a cause of mortality in poultry,^1^ ALV can mutate much easier than other avian viruses.^1,2,3^ ALVs isolated from chickens are divided into six subgroups (A–J) on the basis of the differences in the envelope glycoproteins.^1^ More recently, some ALV isolates from indigenous chicken breeds in East Asia were found to be distantly related to all previously described subgroups; therefore, a novel ALV subgroup (subgroup K) has been suggested.^4^ ALV subgroups exhibit considerable genetic diversity, which results from the high mutation rate of reverse transcriptase, viral genomic recombination, and selection pressures from cell-mediated immune responses.^2^ Although there have been many studies of ALV in commercial layer and meat-type chickens,^5,6^ only limited information regarding the epidemiology and pathogenicity of ALV among chickens indigenous to China is available.

To this end, we performed a large-scale seroepidemiological survey of ALV among several breeder farms in China between 2008 and 2010. Our study identified ALV-A, ALV-B, and ALV-J infections in six breeds of local Chinese ‘yellow' chickens, and only one breed was negative for antibodies to ALV-A/B or ALV-J. Of the 28 investigated chicken breeds indigenous to the six provinces (Shandong, Guangdong, Guangxi, Jiangsu, Anhui, and Hainan) in China, 22 were positive for ALV-A/B antibodies and 23 were positive for ALV-J antibodies. Overall, the majority of the investigated breeds were positive for both ALV-A/B and ALV-J antibodies. Furthermore, only one breed was negative for antibodies to ALV-A/B or ALV-J. These results suggest that ALV infections have become widespread in most chicken breeds indigenous to China.

In addition, a total of 270 clinical samples from chickens (including tumors, whole blood, and eggs) were collected from three provinces (Shandong, Zhejiang, and Jiangsu) in China from 2011 to 2014. These chickens belonged to 22 indigenous species. ALV isolation and identification in a DF-1 cell culture was performed as previously described.^7^ In brief, after an incubation period of seven to ten days, the cell lysates were prepared for ALV group-specific antigens (p27). The positive samples were utilized for the extraction of viral RNA, which was then used for the detection of ALV through reverse transcription polymerase chain reaction.

A total of 46 ALV strains were successfully isolated, cloned, and sequenced using primers designed for ALV. A total of 81 *gp85* gene sequences (including 46 sequences that were newly sequenced in this study and 35 reference sequences from GenBank) of ALV were used for phylogenetic analysis. Our results showed that the 46 recently identified sequences belonged to four ALV subgroups, ALV-A (*n* = 4, 8.7%), ALV-C (*n* = 2, 4.4%), ALV-J (*n* = 18, 39.1%), and ALV-K (*n* = 22, 47.8%), which indicated a high genetic diversity of ALV among chickens indigenous to China (Figure 1).

A sequence comparison analysis revealed that the nucleic acid homology of the *gp85* gene, on which subgrouping is based, ranged from 92.5% to 100% among the 22 ALV-K isolates, and the range was only 77.2%–85.7% when compared to the homologous sequences of ALV subgroups A, B, C, D, and E, which was significantly lower than the *gp85* homology observed within the common chicken subgroups A (89.7%–99.1%), B (91.3%–98.8%), and E (98.0%–99.4%). The *gp85* homology between these strains and subgroup J ranged from only 37.4% to 40.5%. As shown in Figure 1, subgroup J formed an independent, monophyletic clade, compared to the non-J subgroups. All of the viruses isolated from diseased chickens with tumors belonged to ALV-J.^8,9,10^ In contrast, ALV-A, ALV-C, and viruses belonging to the new subgroup K were isolated from clinically healthy chickens. Therefore, the pathogenicity of ALV-J was relatively high among the indigenous chicken breeds.^11^ Most notably, ALV-K had a very high percentage among the ALVs isolated from Chinese indigenous breed chickens but was never isolated and identified from imported breeds of white meat-type chickens and layers over the last 30 years. As suggested, this might be a novel subgroup that exists in local chicken breeds in East Asia.

China is rich in genetic resources related to chickens, and there are many indigenous breeds scattered throughout the country (that yield approximately four billion birds each year). Firstly, genetic and economic losses from ALV-associated diseases are characterized by mortality, reduced egg production, and immunosuppression. Secondly, the diversity of chickens in China and differences in their growth rate provide a good environment for the spread of ALV such as ALV-K. These ALV variants could lead to severe ecological damage due to infection of layer and meat-type chickens. Thirdly, the most important concern is the possibility that a genetic recombinant between the most popular subgroups ALV-J and ALV-K will appear and threaten future chicken flocks similar to the occurrence of highly pathogenic ALV-J that was recently induced into Chinese indigenous breeds and ALV-K, which is native and adapted to the indigenous breeds. Therefore, effective prevention and elimination measures against ALV infections should be implemented as soon as possible.

## Figures and Tables

**Figure 1:**
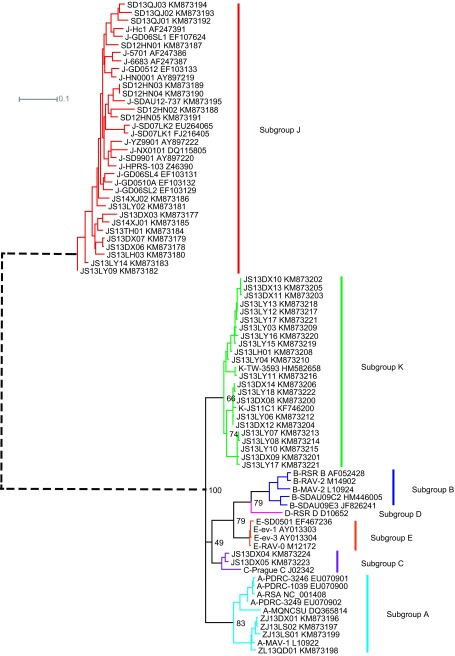
A phylogenetic tree for the *gp85* gene sequences of avian leukosis virus (ALV) isolated from chicken breeds indigenous to China and compared with ALV reference strains of different subgroups. The phylogenetic tree was constructed using MEGA software (version 5.1; www.megasoftware.net) with the neighbor-joining method. The bootstrap values were calculated with 1000 replicates of the alignment. The bootstrap values for major ALV subgroups are shown. All of the reference sequences were acquired from the GenBank database (www.ncbi.nlm.nih.gov/genbank). JS: isolated from Jiangsu province; ZJ: isolated from Zhejiang province; SD: isolated from Shandong province. DX: isolated from Dongxiang Blue-shelled chickens. LH: isolated from Wenshang Luhua chickens. LY: isolated from Langya chickens. TH: isolated from Taihewugu chickens. QJ: isolated from Partridge Shank chickens. QD: isolated from Qiandongnan chickens. LS: isolated from Longsheng Feng chickens. DXH: isolated from Dongxiang Black chickens. The GenBank accession numbers of the sequences are shown after the name of the virus. The scale bar indicates the nucleotide substitutions per site.
